# miRNA-Orchestrated Fibroinflammatory Responses in Heart Failure with Preserved Ejection Fraction: Translational Opportunities for Precision Medicine

**DOI:** 10.3390/diagnostics15182286

**Published:** 2025-09-09

**Authors:** Maria Andreea Micu, Dan Alexandru Cozac, Alina Scridon

**Affiliations:** 1 Emergency Institute for Cardiovascular Diseases and Transplantation of Targu Mures, 540136 Targu Mures, Romania; micu.andreea99@yahoo.com; 2 Physiology Department, George Emil Palade University of Medicine, Pharmacy, Science, and Technology of Targu Mures, 540142 Targu Mures, Romania; alina.scridon@umfst.ro; 3 Doctoral School of Medicine and Pharmacy, George Emil Palade University of Medicine, Pharmacy, Science, and Technology of Targu Mures, 540142 Targu Mures, Romania; 4 Center for Advanced Medical and Pharmaceutical Research, George Emil Palade University of Medicine, Pharmacy, Science, and Technology of Targu Mures, 540142 Targu Mures, Romania

**Keywords:** biomarkers, fibrosis, heart failure with preserved ejection fraction (HFpEF), micro ribonucleic acids (miRNAs)

## Abstract

Heart failure with a preserved ejection fraction (HFpEF) accounts for nearly half of all heart failure cases. It continues to impose a significant global cardiovascular burden due to its rising prevalence, complex pathophysiology, and limited treatment options. The absence of effective disease-modifying therapies is primarily attributable to the complex and heterogeneous pathophysiology underlying HFpEF. The hallmark of HFpEF is systemic inflammation, mostly originating from extracardiac comorbidities, which initiates and sustains the process of myocardial fibrosis, resulting in diastolic dysfunction. Recent evidence has identified specific micro ribonucleic acids (miRNAs) as key regulatory molecules in this inflammation–fibrosis cascade. Particularly, miR-21 and miR-29 play a central role in modulating these pathological processes by regulating the post-transcriptional expression of genes involved in inflammation, cardiac fibrosis, and remodeling. The inflammation-fibrosis axis in HFpEF offers multiple therapeutic opportunities ranging from direct anti-fibrotic strategies to the modulation of inflammation and fibrosis-related miRNA signatures. Such targeted approaches, especially miRNA modulation, hold potential to disrupt fundamental molecular mechanisms driving disease progression, moving beyond conventional HFpEF management. This narrative review explores the roles of miRNAs in modulating inflammation and fibrosis in HFpEF, critically assesses their potential as diagnostic and prognostic biomarkers, and evaluates their therapeutic application. Given the urgent clinical need for efficient HFpEF treatment strategies, understanding miRNA-mediated regulation of the inflammation–fibrosis axis is essential for developing personalized, mechanism-based therapies for HFpEF that could fundamentally change the HFpEF management paradigm.

## 1. Introduction

Heart failure (HF) represents a progressive clinical syndrome with a substantial global epidemiological impact, affecting more than 60 million people worldwide [1]. Heart failure with a preserved ejection fraction (HFpEF) constitutes approximately one-half of all HF cases, though precise prevalence estimates vary across populations and diagnostic criteria [2]. Despite preserved systolic function, HFpEF contributes significantly to cardiovascular morbidity and mortality, with hospitalization rates comparable to those observed in heart failure with a reduced ejection fraction (HFrEF) and even higher mortality rates [3]. With advancing global life expectancy and improved diagnostic capabilities, the incidence of HFpEF has demonstrated a consistent upward trajectory, a trend projected to continue [4]. Due to the pathophysiologic heterogeneity regarding the onset and evolution of HFpEF, there has been no proven, unequivocally effective medical treatment to reverse or at least significantly alleviate cardiac function [5]. This therapeutic challenge is partially caused by fundamental mechanistic differences between HFpEF and HFrEF. While HFrEF typically involves primary cardiac dysfunction leading to secondary inflammatory responses, HFpEF demonstrates a distinctive pathophysiological paradigm wherein systemic inflammation, predominantly driven by extracardiac comorbidities, precedes and subsequently impairs cardiac function. Nevertheless, emerging research has focused on the modulation of the assumed key mediators underlying HFpEF pathophysiology. Chronic inflammation has been identified as a fundamental component of HFpEF physiopathology and represents a potential therapeutic target, particularly through inflammasome modulation [6].

Micro ribonucleic acids (miRNAs) have emerged as a significant area of investigation in molecular genetics since their discovery in 1993 [7]. These single-stranded, endogenously derived, short non-coding RNA (ncRNA) molecules, typically comprising approximately 22 nucleotides, function as post-transcriptionally regulators that either promote target mRNAs cleavage or just suppress translation [8,9]. Optimal utilization of miRNAs as prognostic biomarkers, therapeutic targets, or diagnostic tools requires a comprehensive understanding of miRNA turnover kinetics and the context-dependent molecular mechanisms governing miRNA-mediated gene expression regulation [7,10]. Multiple miRNAs have been associated with distinct HF phenotypes, demonstrating differential expression patterns in both early- and end-stage HF. Nevertheless, clinical evidence establishing a correlation between specific miRNA expression profiles and the development of particular HF phenotypes remains an active area requiring further investigation [11].

Multiple predisposing factors that influence disease progression, particularly regarding structural cardiac changes, are encompassed within HFpEF. Therefore, a comprehensive understanding of molecular markers associated with inflammation and fibrosis in this specific context may provide the foundation for targeted, effective therapeutic approaches to this heterogeneous pathological entity. This review aims to elucidate the potential roles of miRNAs in the HFpEF prediction and diagnosis, explore the feasibility of therapeutic miRNA modulation as a means to reverse underlying pathophysiological processes, and evaluate their prognostic utility in clinical practice.

A comprehensive literature search was conducted in PubMed, Scopus, and Web of Science using the keywords ‘biomarkers’, ‘fibrosis’, ‘heart failure with preserved ejection fraction (HFpEF)’, ‘micro ribonucleic acids (miRNAs)’, and specific miRNAs, such as miR-21, miR-29, and miR-223. We also manually searched reference lists and relevant reviews, and we selected eligible articles based on their relevance to the topic. A summary of the article selection process for this particular narrative review is depicted in the following table (Table 1).

## 2. Chronic Inflammation and HFpEF

**Figure 1:**
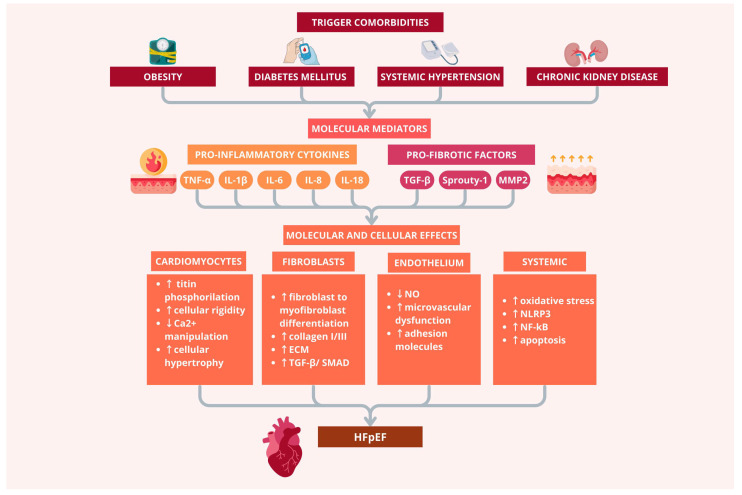
Complex inflammatory and profibrotic pathways contributing to HFpEF development. Triggering comorbidities such as obesity, diabetes mellitus, systemic hypertension, and chronic kidney disease activate molecular mediators, including pro-inflammatory cytokines: tumor necrosis factor α (TNF-α), interleukin 1β (IL-1β), interleukin 6 (IL-6), interleukin 8 (IL-8), interleukin 18 (IL-18) and pro-fibrotic factors: transforming growth factor β (TGF-β), Sprouty-1 and matrix metalloproteinase 2 (MMP2). These mediators cause molecular and cellular effects in cardiomyocytes, fibroblasts, endothelium, and systemic tissues, resulting in increased stiffness, fibrosis, inflammation, and oxidative stress. Collectively, these changes contribute to HFpEF onset.

The pathophysiological paradigm of HFpEF, characterized by systemic inflammation preceding cardiac dysfunction, fundamentally distinguishes it from other HF phenotypes. In HFpEF, the constellation of age-related comorbidities—including diabetes mellitus, obesity, systemic hypertension, and chronic kidney disease—establishes a state of chronic low-grade systemic inflammation that subsequently targets the myocardium (Figure 1) [12].

This comorbidity-driven inflammatory link represents a critical mechanistic pathway through which extracardiac conditions ultimately compromise cardiac structure and function. Unlike the acute inflammatory responses observed during myocardial injury (such as myocardial infarction), chronic inflammation in HFpEF is characterized by sustained, low-level activation of inflammatory pathways without definite resolution. This persistent inflammatory state involves continuous activation of circulating immune cells, including monocytes, T-lymphocytes, and neutrophils, which infiltrate the myocardium and establish local inflammatory foci. The chronicity of this process prevents the typical transition from pro-inflammatory to anti-inflammatory phases that characterizes normal tissue repair, resulting in sustained myocardial damage and progressive functional deterioration [6,13,14].

The molecular signature of chronic inflammation in HFpEF is characterized by persistent elevation of key pro-inflammatory cytokines, particularly interleukin (IL)-1β, tumor necrosis factor-α (TNF-α), and IL-6 (Figure 1) [14,15]. These molecules have been demonstrated to play pivotal roles in the pathogenesis of cardiac hypertrophy and diastolic dysfunction, via mitogen-activated protein kinases (MAPKs) and nuclear factor kappa-light-chain-enhancer of activated B cells (NF-κB) pathways. Clinical studies have consistently demonstrated elevated circulating levels of these inflammatory mediators in HFpEF patients compared to healthy controls, with concentration correlating with disease severity [13,14].

In the context of HFpEF, TNF-α exerts particularly detrimental effects on cardiac structure and function. Beyond its direct effects on cardiomyocyte hypertrophy and contractile dysfunction, TNF-α promotes extracellular matrix (ECM) remodeling and induces cardiomyocyte apoptosis (Figure 1). The cytokine’s effects are mediated through differential activation of TNF receptors (TNFR1 and TNFR2), with TNFR1 activation predominantly mediating deleterious effects while TNFR2 activation may confer cardioprotective properties [13,16]. Additionally, TNF-α disrupts calcium homeostasis by downregulating calcium-regulating genes, leading to sarcoplasmic reticulum dysfunction that contributes to diastolic stiffness and impaired relaxation, hallmark features of HFpEF (Figure 1). IL-1β contributes to HFpEF pathophysiology through similar mechanisms affecting calcium homeostasis, producing delayed and prolonged myocardial contractility, and is a trigger for cardiomyocyte pyroptosis [16,17]. IL-6, while capable of acutely enhancing cardiac contractility, promotes maladaptive cardiac hypertrophy when chronically elevated, and it also reduces the phosphorylation of titin, thus increasing cardiomyocyte stiffness—a key pathophysiological feature underlying diastolic dysfunction in HFpEF (Figure 1) [13,16].

The chronic inflammatory state in HFpEF also disrupts angiogenesis, which plays a crucial role in disease progression. Pro-inflammatory cytokines, including transforming growth factor (TGF)-β, IL-6, IL-8, IL-1, and TNF-α, possess dual roles as both inflammatory mediators and angiogenic regulators [18]. When the angiogenic response becomes maladaptive, as in the HFpEF pathophysiological mechanisms, this will contribute to abnormal vascular remodeling and impaired coronary microvascular function [18]. Sanders-van Wijk et al. report that inflammatory mediators (including TNFR 1, urokinase plasminogen activator receptor, insulin-like growth factor-binding protein 7, and growth differentiation factor 15) account for 13–41% of the association between comorbid burden and echocardiographic abnormalities [19]. Research has previously shown that inflammatory markers such as IL-6 and TNF–related proteins are associated with diastolic dysfunction and clinical severity [20,21]. Additionally, Carris et al. document that elevated IL-2 levels predict incident HFpEF [22]. The complex network of cellular interactions contributing to HFpEF development is depicted in Figure 1.

## 3. Mechanisms of Myocardial Fibrosis in HFpEF

Chronic systemic inflammation, triggered by a cluster of metabolic comorbidities, leads to the persistent elevation of pro-inflammatory cytokines. This sustained inflammatory state promotes a cascade of pathophysiological changes that culminate in myocardial fibrosis and the typical HFpEF phenotype [14,23,24]. Interstitial and perivascular fibrosis are the two histological types of reactive fibrosis that occur in HFpEF, involving fibrotic transformations without myocyte apoptosis [25]. From a cellular point of view, in HFpEF, the myocytes are enlarged and with increased stiffness, due to the shift in the collagen isoform and high collagen synthesis [26]. There is a certain correlation between increased fibrillar collagen matrix content and high myocardial rigidity, which is a significant contributor to HFpEF pathogenesis [27].

Chronic inflammation induces coronary microvascular endothelial activation, which represents a critical early step in the inflammation-fibrosis pathway. This endothelial dysfunction is characterized by upregulation of vascular cell adhesion molecules (VCAMs), increased endothelial membrane permeability that allows the transformation of infiltrating monocytes into TGF-β-secreting macrophages, causing collagen secretion by fibroblasts. Simultaneously, endothelial activation leads to reduced nitric oxide production, coupled with enhanced reactive oxygen species (ROS) production [27].

However, the transition from systemic inflammation to myocardial fibrosis involves several interconnected molecular pathways that create a self-perpetuating cycle of tissue remodeling. As previously mentioned, IL-1β promotes the expression of matrix metalloproteinases and the secretion of chemokines, while maintaining low α-smooth muscle actin levels, thereby creating an environment conducive to matrix turnover [27]. TNF-α enhances TGF-β induction and upregulates lysyl oxidase expression, facilitating collagen cross-linking and establishing the structural foundation for increased myocardial stiffness [20]. Moreover, inflammation impairs the unfolded protein response (UPR), leading to cytoplasmic accumulation of misfolded proteins such as titin or troponin. This may explain why patients with HFpEF typically have elevated serum troponin levels with no apparent reason for cardiomyocyte apoptosis [27]. An important component of HFpEF is increased left ventricular incompliance and stress maladaptation that can be due to a shift in the titin isoform, a decrease in the N2BA/N2B ratio (two distinct titin isoforms coexpressed in the human myocardium, N2B being stiffer [28]) caused by increased hemodynamic load, hypeophosphorylation, and oxidative stress enabled by inflammation [27]. Titin is a giant sarcomeric protein that functions as a structural scaffold for thick filament organization and a bidirectional molecular spring. By storing potential energy during myocardial contraction, titin generates restorative force and passive tension, aiding sarcomere re-extension that could resist overstretching, thereby influencing cardiac relaxation and diastolic mechanics [29]. Nevertheless, fibroblasts may also confer a pro-inflammatory state by secreting cytokines and chemokines after being triggered by damage-associated molecular patterns (DAMPs) [14]. IL-1β, TNF-α, and activation of toll-like receptors (TLRs) could induce a pro-inflammatory fibroblast phenotype, which contributes to this loop of inflammation [14]. The TGF-β signaling axis represents the critical convergence point between inflammation and fibrosis, serving as the primary mediator of fibroblast-to-myofibroblast transdifferentiation. This transformation process involves α-smooth muscle actin incorporation into the cellular cytoskeleton and enhanced collagen synthesis while simultaneously inhibiting matrix degradation through metalloproteinase suppression. The mechanical tension generated by contractile myofibroblasts creates a positive feedback loop that stimulates further TGF-β secretion, establishing a mechanically driven perpetuation of the fibrotic process [13,14,17,26,30,31].

The renin–angiotensin–aldosterone system (RAAS) plays a crucial amplifying role in this inflammation-fibrosis link [30]. Inflammatory cytokines upregulate the expression of angiotensin II type 1 receptors (AT1R) on cardiac fibroblasts, thereby amplifying angiotensin II (Ang II)-mediated fibroblast responses that promote fibrotic remodeling of the myocardium [13]. Ang II is a key pro-fibrotic and hypertrophic mediator in the heart, and it acts via the AT1 receptor to stimulate TGF-β signaling, which determines Smad 2/3 phosphorylation and enhanced transcription of the ECM components and pro-inflammatory molecules. Moreover, RAAS activation promotes myofibroblastic differentiation, collagen production, and oxidative stress, collectively contributing to cardiomyocyte hypertrophy, collagen deposition, and pro-fibrotic cascade—core processes underlying the pathophysiology of HFpEF [32,33].

The inflammation-fibrosis relationship in HFpEF provides multiple therapeutic intervention points that extend beyond traditional HF management approaches. Direct anti-fibrotic approaches focusing on TGF-β pathway inhibition and matrix metalloproteinase modulation represent emerging therapeutic directions, and metabolic optimization addressing underlying comorbidities driving systemic inflammation may provide foundational benefits.

## 4. miRNAs in HFpEF-Associated Chronic Inflammation

The intricate molecular crosstalk between inflammation and fibrosis in HFpEF is further modulated by epigenetic regulatory mechanisms, particularly miRNAs [11]. The dysregulation of specific miRNA profiles in HFpEF creates a permissive environment for sustained inflammation and progressive fibrosis, as these regulatory molecules can simultaneously target multiple components of the TGF-β signaling pathway, pro-inflammatory cytokine networks, and fibroblast differentiation programs [20]. Moreover, the bidirectional nature of miRNA regulation allows for both pro-inflammatory and anti-inflammatory miRNAs to be dynamically modulated in response to the metabolic and hemodynamic stresses characteristic of HFpEF, creating a complex regulatory network that ultimately determines the balance between adaptive and maladaptive cardiac remodeling.

Understanding the specific miRNA signatures associated with chronic inflammation in HFpEF provides crucial insights into the molecular mechanisms underlying disease progression and offers potential therapeutic targets for interrupting the pathological inflammation-fibrosis cycle at the post-transcriptional level.

**Figure 2:**
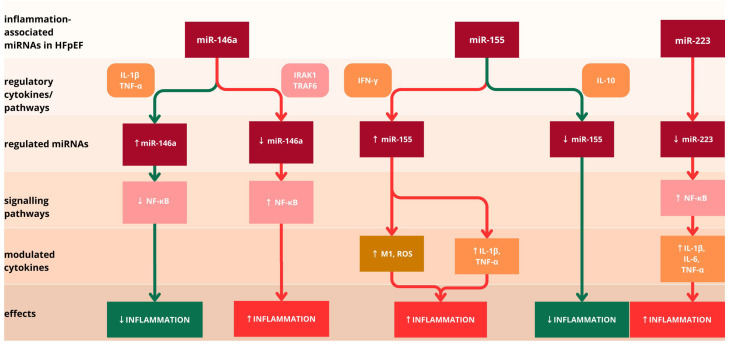
The implication of miR-146a, miR-155, and miR-223 in myocardial inflammation modulation. Red arrows represent pathways driving pro-inflammatory responses, whereas green arrows denote those associated with attenuation of inflammation. miR-146a is up-regulated by pro-inflammatory cytokines interleukin 1β (IL-1β) and tumor necrosis factor α (TNF-α), which inhibits the nuclear factor kappa-light-chain-enhancer of activated B cells (NF-κB) signaling pathway, resulting in a reduction in inflammation. Conversely, decreased miR-146a leads to sustained NF-κB activation via interleukin-1-associated-kinase-1 (IRAK1) and tumor necrosis factor receptor-associated factor 6 (TRAF6), leading to enhanced inflammation. miR-155 promotes the activation of pro-inflammatory type-1 macrophage (M1) and reactive oxygen species (ROS) production, as well as secretion of IL-1β and TNF-α, thereby amplifying the inflammatory response. miR-155 expression is suppressed in the presence of interleukin 10 (IL-10), resulting in reduced inflammatory signaling. The downregulation of miR-223 leads to NF-κB activation, which drives the upregulation of pro-inflammatory cytokines IL-1β, interleukin 6 (IL-6), and TNF-α, ultimately contributing to increased inflammation. The regulatory roles of miR-146a, miR-155, and miR-223 in modulating inflammatory pathways are highly relevant to HFpEF progression.

miR-146a functions as a critical anti-inflammatory and antifibrotic regulator, with established prognostic significance in cardiovascular pathology [8]. Clinical evidence demonstrates its association with increased cardiovascular mortality following acute coronary syndromes, major adverse cardiovascular events, HF, and left ventricular remodeling [8,34]. The regulatory mechanism of miR-146a involves stimulus-responsive expression patterns triggered by pro-inflammatory cytokines, such as IL-1β and TNF-α [35]. Upon activation, miR-146a exerts post-transcriptional suppression of NF-κB pathway components, thereby attenuating inflammatory signaling cascades (Figure 2) [32]. Conversely, a deficiency of miR-146a results in the elevated expression of inflammatory cytokines IL-1β, IL-18, and other inflammasome activation markers in macrophages and may also determine NF-κb activation via the interleukin-1-associated-kinase-1 (IRAK1) and the tumor necrosis factor receptor-associated factor 6 (TRAF6) (Figure 2) [8,36]. Additionally, miR-146a can function in synergy with miR-21, determining reduced apoptosis and inhibition of autophagy in cardiomyocytes after an ischemic event [37]. Contradictory evidence suggests that it could also have pro-inflammatory properties, as extracellular miR-146a-5p can induce myocardial inflammation in murine hearts via the toll-like receptor 7 (TLR7) [38]. However, therapeutic modulation aimed at restoring miR-146a function could address both the inflammatory burden and fibrotic progression in HFpEF.

miR-155 represents one of the most abundantly expressed pro-inflammatory miRNAs with multiple roles in cardiovascular pathology [8]. It has fundamental implications in epicardial development and myofibroblast density modulation and confers protection against cardiomyocyte apoptosis [34]. miR-155 is crucial for T helper cell differentiation and also plays a pivotal role in B cells and dendritic cells’ functional maturation [39]. Within the context of myocardial inflammation, miR-155 upregulation triggers pro-inflammatory type-1 macrophage (M1) polarization, which determines pro-inflammatory factors and ROS synthesis [40]. Given miR-155’s pro-inflammatory role, targeted inhibition could reduce M1 macrophage polarization and associated inflammatory mediator production, potentially improving the chronic inflammatory state characteristic of HFpEF [41]. Furthermore, miR-155 upregulation in murine M1 macrophages stimulated by lipopolysaccharides and interferon-gamma determined increased expressions of pro-inflammatory genes such as TNF-α and IL-1β, along with their corresponding nuclear proteins (Figure 2) [42]. Lipopolysaccharides regulate miR-155 expression through activation of the myeloid differentiation primary response protein 88 (Myd88) and the TIR-domain-containing adapter-inducing interferon-β (TRIF) signaling pathways, while IL-10 is implicated in its downregulation (Figure 2) [8].

miR-223 is predominantly expressed in cardiomyocytes and functions as a negative regulator of neutrophil activation and chemotactic responses [34,43]. It is highly expressed during granulocyte differentiation, and while not required for granulocyte differentiation, it is critically essential for proper granulocyte maturation [44,45]. Its downregulation promotes IL-1β and IL-6 production and determines further enhancement of NF-κB activation, supplementary increasing IL-6 and TNF-α expression and thereby contributing to systemic and myocardial inflammation, central features of HFpEF (Figure 2) [46]. Despite the complex and multifaceted roles of miR-223 in myocardial inflammation, there is considerable interest in investigating its dual function concerning its clinical significance in HF. According to Liu et al., miR-223 primarily contributes to adverse cardiac remodeling and progression to HF after myocardial infarction by enhancing fibrotic signaling, but clinical research regarding the implications of miR-223 in specifically HFpEF is limited [47]. The regulatory functions of miR-146, miR-155, and miR-223 are illustrated in Figure 2.

## 5. miRNAs-Mediated Myocardial Fibrosis

miR-21 is both cardiomyocyte-enriched and fibroblast-derived miRNA with utmost importance in the regulation of cardiac fibroblast proliferation and myocardial fibrosis [34,48]. Albeit initial research on miR-21 was approached from the perspective of the spectrum of neoplastic and autoimmune pathologies, it was later demonstrated that it shows a regular pattern of overexpression and is upregulated in HF [10,37,48]. Evidence suggests that the overexpression of miR-21 determines heightened myocardial fibrosis by activating the TGF-β-pathway-mediated migration of cardiac fibroblasts and the fibroblast-to-myofibroblast differentiation [48,49]. Within the context of myocardial fibrosis, the suppression of Smad7 leads to the upregulation of Smad2 and Smad3 phosphorylation, thereby promoting subsequent processes involved in the synthesis of ECM [48]. Additionally, miR-21 directly targets Sprouty-1 in cardiac fibroblasts (a protein encoded by the SPRY1 gene in humans, associated with the regulation of cellular senescence), which leads to increased fibroblast density and survival, as shown in a postischemic rat HFrEF model [50,51]. In HF specimens, there were enhanced levels of miR-21 in cardiac fibroblasts, which determined increased fibroblast survival and hypersecretion of ECM, leading to interstitial fibrosis by modulating the extracellular signal-regulated kinase (ERK)-mitogen-activated protein (MAP) kinase signaling pathway [52,53]. The latter was also noticeable in the cardiac remodeling after a primary ischemic injury in the later stages of HF onset [52]. The inhibition of miR-21 through a specifically synthesized antagomir was proved to diminish hypertrophy and fibrosis in an in vivo mouse model of pressure-overload-induced cardiac disease [54]. However, the precise implication of miR-21 in the onset and evolution of cardiac hypertrophy continues to be debatable [10]. Furthermore, increased expression of miR-21 is associated with aging-specific cardiac hypertrophy post Ang II supplementation, emphasizing the fact that Ang II effects are dependent on the miR-21 levels [55]. Regarding the early stages of myocardial infarction, miR-21 is thought to be cardioprotective by decreasing apoptosis induction [56]. However, there has also been experimental evidence on a rat model of streptozotocin-induced diabetic cardiomyopathy, implying that miR-21 determines pyroptosis of the cardiac fibroblasts through the inhibition of the androgen receptors in diabetic fibrotic myocardium [57].

The miR-29 family encodes a series of ECM proteins involved in fibrosis, such as collagen, fibrillin, and elastin, hence acting as a regulator in the process of myocardial fibrosis [52,58]. miR-29 downregulation induces expression of key fibrotic proteins, including elastin, fibrillin 1, collagen type I, α1 and α2, and collagen type III, α1, enhancing its role in cardiac fibrosis via direct effects on the matricellular proteins [24,59]. This mechanism is particularly relevant to HFpEF, where diffuse myocardial fibrosis directly correlates with diastolic dysfunction severity. Evidence suggests that miR-29 has an active role in the TGF-β/Smad pathway suppression via the modulation of TGFβ2 and matrix metalloproteinase 2 (MMP2). Contrarily, Smad3 downregulates miR-29 by binding to its promoter, thus conferring a profibrotic status [60]. Besides its role in collagen regulation in the myocardial extracellular matrix, miR-29 has been proven to have implications in HFpEF via myocardial hypertrophy signaling pathways, calcium handling, the function of the endoplasmic reticulum, UPR, and oxidative stress [61]. In patients diagnosed with hypertrophic cardiomyopathy, miR-29a, a member of the miR-29 family, was the only miRNA to be associated with both left ventricular hypertrophy and myocardial fibrosis and was positively correlated with the interventricular septum diameter [53]. miR-29a amelioration of myocardial hypertrophy is explained through its effect on the nuclear receptor peroxisome proliferator-activated receptor delta (PPARD) and through downregulation of the atrial natriuretic factor [62]. Moreover, miR-29a and miR-29b are thought to be prognostic biomarkers in the postischemic HFrEF evolution [63]. miR-29 overexpression correlates with multiple cardiovascular conditions, including cardiomyopathy, myocardial fibrosis, atrial fibrillation, atherosclerosis, coronary heart disease, and arterial aneurysms, indicating its broad therapeutic potential across HFpEF [63].

miR-208a and miR-208b are intronic miRNAs transcribed from the Myh6 and Myh7 genes, which encode the α-isoform of myosin heavy chain (α-MHC), the preponderant MHC isoform in the adult heart, and the β-isoform of the myosin heavy chain (β-MHC), respectively [64]. In the hypertensive HF phenotype, the myosin isoform shifts from α-MHC to β-MHC, and concordant increased Myh7 expression is believed to be one of the cornerstones of the pathogenic remodeling of the myocardium [64,65]. As a whole, overexpression of the miR-208 family was incriminated in the progression of myocardial hypertrophy, with the overexpression of miR-208b and downregulation of α-MHC [52]. Despite its fundamental role in cardiac progenitor cell population differentiation in the embryonic stages of organ development, increased expressions of miR-208b did not augment cardiomyocyte proliferation, but rather had an impact on hypertrophy and the onset of atrial fibrillation and HF [34]. It was further established that under mechanical stretch conditions, TGF-β induces the expression of miR-208a, which subsequently promotes the expression of endoglin and stimulates the formation of collagen I. This sequence of events facilitates the differentiation of cardiomyocytes into myofibroblasts, ultimately resulting in cardiac fibrosis, hence exhibiting the active role of miR-208 in cardiac fibrosis [66].

miR-133a is an anti-fibrotic regulator by directly targeting the Collagen1A1 gene, which enhances myoblast proliferation and regulates sarcomere formation, cardiomyocyte structure, proliferation, and cardiac conduction [34,67]. Its implications in hypertrophic cardiomyopathy and HF can be extrapolated from its reduced expression and downregulation in this specific population [10,37].

Members of the miR-15 family play critical roles in the cardiovascular system by inducing apoptosis, suppressing mitosis, and reducing cardiomyocyte proliferation. Their contribution to cardiac hypertrophy regulation has been objectified by administering anti-miR-15 therapy to murine models of cardiac ischemia–reperfusion injury to downregulate hypertrophic signaling, reduce infarct size and adverse cardiac remodeling, and enhance cardiac function [10,34]. A summary of the aforementioned miRNAs’ expression profile in HFpEF can be found in Table 2.

## 6. Interaction Between miRNAs Involved in Cardiac Inflammation and Fibrosis

### 6.1. The Duality of miRNAs in Modulating Inflammatory and Fibrotic Pathways

The networks of shared regulation of the mechanisms responsible for the onset of HFpEF may involve the activation of mediators common to both inflammation and fibrogenesis. Galectin-3 serves as a critical inflammatory factor that promotes cardiac and tissue fibrosis, with increased levels being associated with both inflammation and fibrosis. The evidence shows that inhibition of galectin-3 function strongly reduces expression of pro-inflammatory mediators, such as IL-6, IL-1β, IL-23, and P19, and upregulates IL-10, IL-12, TLR/NLR-pathways in dendritic cells and monocytes, thereby inhibiting the development of Th17/T2 cells and innate immunity [68]. However, these findings cannot be fully translated to HFpEF pathophysiology, emphasizing the complexity of these molecules across different diseases.

Recent evidence suggested that dysregulated miRNAs in myocarditis show phase-dependent changes and correlate with viral infection, immune status, fibrosis, destruction of cardiomyocytes, arrhythmias, and cardiac function, demonstrating the temporal complexity of miRNA networks in disease progression [69].

Galectin-3 is tightly regulated by several miRNAs—including miR-199a, miR-27b, miR-204-5p, miR-335, miR-1, miR-21, and miR-214—whose expression patterns correlate with pathological myocardial remodeling processes such as hypertrophy, ischemia/reperfusion injury, and HF. Interactions within the miRNA/Galectin-3 axis further modulate galectin-3 expression, suggesting that targeting this regulatory network could offer promising diagnostic and therapeutic strategies against fibrosis-driven cardiac dysfunction [70].

**Figure 3:**
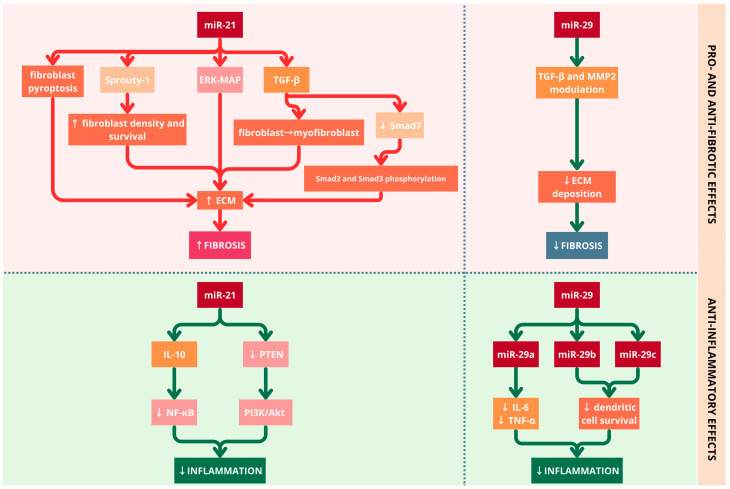
The duality of miR-21 and miR-29 in the etiopathogenesis of HFpEF. Red arrows represent pathways driving pro-inflammatory and pro-fibrotic responses, whereas green arrows denote those associated with attenuation of inflammation and fibrosis. miR-21 has anti-inflammatory effects by promoting interleukin 10 (IL-10) expression, reducing nuclear factor kappa-light-chain-enhancer of activated B cells (NF-κB) signaling, and by phosphatase and tensin homolog (PTEN) downregulation via the phosphoinositide 3-kinase/protein kinase B (PI3K/Akt) pathway. miR-21 promotes fibroblast survival, extracellular signal-regulated kinase-mitogen-activated protein (ERK-MAP) activation, transforming growth factor β (TGF-β) signaling, and myofibroblast differentiation, ultimately increasing extracellular matrix (ECM) deposition and fibrosis. miR-29 reduces interleukin 6 (IL-6) and tumor necrosis factor α (TNF-α) levels (via miR-29a) and limits dendritic cell survival (via miR-29b and miR-29c), thereby suppressing inflammation. miR-29 is upregulated by Smad3 and attenuates fibrosis through the inhibition of TGF-β/Smad signaling and ECM accumulation. Through their dual roles in modulating both inflammation and fibrosis, miR-21 and miR-29 shape the cardiac remodeling processes central to HFpEF pathophysiology.

Concurrently, miR-21 modulates inflammatory responses by targeting multiple signaling pathways, including the phosphoinositide 3-kinase/protein kinase B (PI3K/Akt) pathway, through inhibition of the phosphatase and tensin homolog, which has the function of a key brake of the pathway [71]. Moreover, it also intricately modulates the NF-κB pathway through a bidirectional mechanism wherein it suppresses excessive inflammatory responses by inhibiting NF-κB activation and enhancing IL-10 production, and simultaneously it potentiates NF-κB/NLRP3 inflammasome signaling [35]. Activation of the NLRP3 inflammasome causes caspas-1 dependent pyroptosis, leading to IL-1β and IL—18 maturation; these cytokines sustain local inflammation [72]. This dual action contributes to adverse cardiac remodeling and has been implicated in the progression of HF (Figure 3).

In addition to its established role in regulating fibrosis, miR-29a also plays a key role in modulating inflammation. An experimental in vitro study revealed that miR-29a exerts an anti-inflammatory effect by directly targeting the LPL gene, leading to suppression of pro-inflammatory cytokine production, notably decreasing IL-6 and TNF-α secretion [73]. miR-29b and miR-29c act as negative regulators in dendritic cell survival by promoting plasmacytoid dendritic cell apoptosis (Figure 3) [74]. Although primarily a hepatocyte-specific miRNA, miRNA-122 has been proved to exert a dual role by modulating both myocardial inflammation—through its involvement in autophagy, apoptosis, oxidative stress, and hypertrophic signaling pathways—and cardiac fibrosis—via attenuation of TGF-β1 signaling, suppression of MMP2 expression, and promotion of pro-fibrotic remodeling in diabetic cardiomyopathy and aortic valve stenosis [75]. The paradoxical roles of miR-21 and miR-29 in cardiac remodeling are presented in Figure 3.

### 6.2. Circulating miRNAs as Diagnostic Biomarkers

Circulating miRNAs hold strong potential as diagnostic biomarkers in HF due to their remarkable stability and disease-specific expression patterns, with specific examples including miR-208 and miR-150 for cardiac hypertrophy and remodeling, miR-21 for coronary heart disease in the elderly, and miR-19b, miR-423-5p, and miR-92b-5p for various forms of HF [54].

Although miR-208a is exclusively expressed in cardiac muscle, it can be actively secreted into the bloodstream by cardiomyocytes in response to cardiac stress [64]. A study conducted on isoproterenol-treated mouse models demonstrated that plasma miR-208a levels strongly correlate with cardiac troponin I. Despite existing uncertainties regarding whether its upregulation is adaptive or pathological and whether it plays a functional role in cardiac remodeling, the consistent association between miR-208a abundance and myocardial stress positions it as a promising, non-invasive biomarker for diagnosing cardiac dysfunction [64]. Circulating miRNA-21 in both plasma and serum not only escalates in tandem with advancing functional impairment and distinguishes HF patients from healthy individuals with remarkable accuracy, but also portends unfavorable clinical trajectories by associating with diminished survival prospects and, particularly in its serum form, indicating a heightened predisposition to rehospitalization—collectively affirming miRNA-21’s comprehensive value as a non-invasive biomarker for HF assessment and prognostication [76]. Even though there is tangible evidence regarding the correlation between increased serum-specific miRNA values and cardiac dysfunction, we still do not have a clear panel of diagnostic biomarkers that could be implemented to facilitate HFpEF diagnosis.

### 6.3. Relationship Between miRNA Levels and Disease Severity: Prognostic Implications of miRNA Profiling

In addition to the existing challenges in the early diagnosis of HFpEF, there are also significant gaps in its staging and objective prognostic assessment. These limitations underscore the importance of investigating novel prognostic biomarkers, such as miRNA panels. Wang et al. revealed that serum miR-21-5p levels are significantly elevated in patients with HFrEF and progressively increase with higher NYHA functional classes, showing strong positive correlations with established cardiac stress markers such as NT-proBNP, left atrial diameter, and pulmonary pressures, and inverse proportionality with left ventricular ejection fraction and fractional shortening [77]. Moreover, miR-21-5p independently predicts cardiovascular re-hospitalization and mortality, underscoring its value as a prognostic biomarker that reflects both the severity and progression of HFrEF and offers complementary insight alongside conventional indicators [77]. Data from the Japan Collaborative Cohort Study for Evaluation of Cancer Risk demonstrated that high circulating seric levels of miR-21 and miR-29a detected via quantitative real-time reverse transcription polymerase chain reaction were correlated with a higher risk of total death, cancer death, and cardiovascular death in comparison to medium seric levels [78]. Moreover, low serum levels of miR-126 were associated with a higher risk of total death than those with medium levels [78].

Dickinson et al. identified miR-16, miR-20b, miR-93, miR-106b, miR-223, and miR-423-5p as the most significantly altered miRNAs in response to hypertension-induced HF. This specific biomarker panel shows a progressive increase throughout HF and is strongly correlated with levels of circulating BNP and Myh7 expression [65].

Emerging evidence links specific miRNAs to the development of diastolic dysfunction and HFpEF, particularly in the context of diabetes and microvascular injury. The miR-30 family promotes oxidative stress, impaired nitric oxide signaling through altered fatty acid metabolism, and regulates fatty acid metabolism in endothelial cells, as observed in rodent models, contributing to coronary microvascular dysfunction and subsequent HFpEF [79]. Conversely, miR-34a-5p is elevated in diabetic HFpEF, shows greater dysregulation in women and its modulation may reflect both vascular and renal influences in diastolic dysfunction [80]. miR-92a-3p has been identified as a pathogenic driver of HFpEF via endothelial-mesenchymal transition and vascular gene dysregulation [80]. miR-126a, an endothelial-specific miRNA, is reduced in both cardiac tissue and exosomes in diabetic HFpEF, correlating with impaired output, suggesting that loss of this vascular-protective signal exacerbates microvascular rarefaction and diastolic dysfunction [29]. Together, these miRNAs represent mechanistic links between endothelial injury, microvascular dysfunction, and the progression to HFpEF.

A study conducted on a Takotsubo syndrome rat model by Couch et al. attested to the co-overexpression of miR-16 and miR-26a in apical cardiomyocytes after an adrenaline bolus, determining segmental hypokinesia. Thus, they highlighted miR-16 and miR-26’s effect on the increase in the myocardial sensitivity to Takotsubo-like changes induced by adrenaline, and, given their known association with anxiety and depression, they may offer a mechanism by which prior stress primes the heart, heightening the future risk of developing Takotsubo syndrome [81]. Collectively, these findings highlight the prognostic value of miRNAs as HFpEF biomarkers. Their ability to reflect underlying pathophysiological processes offers promising future directions for improving cardiovascular risk stratification beyond traditional markers in patients with HFpEF.

### 6.4. Comparison with Validated Biomarkers for the Heart Failure Spectrum

Comparative studies are essential to evaluate the prognostic and diagnostic value of new biomarkers to established, validated biomarkers in the HF spectrum in order to determine their clinical relevance and reliability. Watson et al. and Guo et al. highlighted miRNAs additive value, especially miR-221, as biomarkers by providing enhanced diagnostic accuracy beyond BNP or NT-proBNP alone. Furthermore, Wong et al. demonstrated that by using a panel of eight circulating miRNAs in conjunction with NT-proBNP, the process of detecting and classification of chronic HF could be facilitated [82]. Arul et al. suggested that miR-210-3p may serve as a complementary marker reflecting the fibrotic stage of HF due to its positive association with galectin-3 [83]. Furthermore, Parvan et al. stated that pooled data support a multi-miRNA panel (miR-328-5p, miR-30c-5p, miR-221-3p, miR-19b-3p) to demonstrate great specificity and sensitivity for distinguishing HFpEF from HFrEF, in some cases outperforming BNP diagnostic value [84]. To a greater extent, Zhang et al. evidence that miR-223-3p has greater specificity than BNP or NT-proBNP by reflecting not only disease severity but also short-term mortality risk, rehospitalization likelihood, and comorbid conditions such as atherosclerosis and renal dysfunction [85]. A combined biomarker approach could significantly improve diagnostic specificity and accuracy compared to NT-proBNP alone, particularly in identifying cases of HFpEF that are often missed by conventional diagnostics [82].

## 7. Potential Therapeutic Approaches

There are currently no miRNA-based therapies approved for clinical use, highlighting the gap between preclinical promise and clinical reality. However, therapeutic strategies involving miRNA mimics and inhibitors (antimirs) show promise in correcting dysregulated miRNA expression, as demonstrated in preclinical models, but their clinical translation remains limited by challenges in targeted delivery, potential off-target effects, and incomplete understanding of their pharmacological profiles [86]. Antagomirs or antimirs are synthetic single-stranded nucleotides that act as competitive inhibitors by specifically binding to target mature miRNAs, blocking their interaction with mRNAs and reducing the effects of miRNA overexpression [10]. Natural miRNAs are stable due to their encapsulation in microvesicles, while exogenous miRNAs require chemical modifications and advanced delivery systems such as antisense oligonucleotides (ASOs), small interfering RNAs (siRNAs), and miRNA sponges to enhance stability, cellular uptake, and therapeutic efficacy [34]. Furthermore, miRNA-based therapies could become conventional only if safety is guaranteed. It involves tailoring delivery strategies to precisely direct therapeutic agents to target tissues and cells at effective concentrations, thereby reducing systemic exposure and preventing accumulation in non-target organs. These strategies minimize off-target effects, lower the risk of immune activation or nonselective activity, and prevent systemic toxicity from unintended biodistribution [87].

Montgomery et al. demonstrated that through therapeutic inhibition of miR-208a via subcutaneous delivery of locked-nucleic-acid-modified ASOs—engineered for in vivo stability, high-affinity binding, and increased specificity—they could achieve selective accumulation in nondividing cardiomyocytes. By obtaining miR-208a silencing, they contributed to pathological cardiac remodeling attenuation and restored diastolic function—evidenced by shortened isovolumic relaxation time and normalized E/A filling ratios—while reducing cardiomyocyte hypertrophy and periarteriolar fibrosis [88]. In cardiomyopathy models, miR-21 inhibition has been shown to attenuate fibrosis and improve cardiac function, yet therapeutic translation is hampered by inconsistent miR-21 expression patterns and its ubiquitous, non-tissue-specific distribution, which raises concerns about off-target effects. To overcome these obstacles, deeper insight into the complex, context-dependent regulation of miR-21 is essential so that future miR–21–directed interventions can be precisely localized and safely deployed [49].

Although no miRNA-based drugs have yet reached clinical practice, numerous trials are exploring miR-21 both as a diagnostic marker and a therapeutic target across diverse conditions, from cancer to genetic kidney disorders [49]. For instance, over the past decade, biopharmaceutical companies have actively pursued miRNA-based therapeutics—exemplified by miravirsen, the first RNA-modified antimiR targeting miR-122 in hepatitis C—by leveraging two principal strategies: antagomiRs to inhibit overactive miRNAs and synthetic mimics to restore those that are underexpressed [49].

Several currently approved cardioactive and non-cardioactive pharmacological agents have been shown to exert beneficial modulatory effects on myocardial inflammation and the progression of cardiac fibrosis [89]. Pirfenidone, an approved anti-fibrotic agent for idiopathic pulmonary fibrosis, has demonstrated anti-inflammatory and cardioprotective effects by reducing TGF-β expression, with promising results from the PIROUETTE phase 2 trial showing a reduction in cardiac fibrosis in HFpEF, warranting further clinical studies to assess its safety and efficacy [89]. While reversing fibrosis remains challenging, sodium-glucose cotransporter 2 inhibitors (SGLT2i), such as dapagliflozin, have emerged as an important therapeutic option for HFpEF. They currently are the only medication to be given a Class I, level A indication for HFpEF patients by the most recent European Society of Cardiology Guidelines, as they reduce HF hospitalization across the preserved EF range [90]. SGLT2i reduces pro-inflammatory mediators (NF-κB, TNF-α, IL-1 β) and suppresses inflammasome activation via NLRP3 modulation, thus alleviating chronic low-grade inflammation contributing to diastolic dysfunction [91]. As aforementioned, miR-21 has similar mechanistic implications. Dapagliflozin exerts benefits by inhibiting TGF-β/Smad signaling, thus ameliorating myocardial fibrosis in a manner common to the physiological implications of miR-29 as previously described [92]. Thereby, modulation of miR-21 and miR-29 pathways could contribute to extending the cardioprotective effects of SGLT2i to other miRNA-based therapies. These findings open awareness for novel therapeutic strategies targeting cardiac remodeling processes.

## 8. Gaps in Knowledge and Future Directions

### 8.1. miRNA Dynamics and Correlation with Cardiac Fibrosis

Fibrosis research is hindered by in vitro limitations of fibroblast differentiation and the lack of specific in vivo fibroblast markers, making targeted investigations difficult. However, emerging technologies like single-cell RNA sequencing have revealed cardiac fibroblast heterogeneity, highlighting the need for future studies to map their temporal dynamics and functional roles in fibrosis, to ultimately lead to more precise cardiac antifibrotic therapies [93].

Another relatively unexplored area of miRNAs is those found in the human pericardial space. Beyond their release into the systemic circulation, miRNAs may also be secreted into other physiological fluids, including pericardial fluid. Pericardial-fluid miRNAs have not yet been explored from a biomarker point of view; however, MMP2 in the pericardial fluid has already proven to be a more sensitive biomarker than its serum counterpart in the context of myocardial remodeling [94]. An interesting approach could represent the discovery of an early-stage HFpEF-specific pericardial fluid miRNA and its modulation in order to limit the pathological progression. Nevertheless, performing routine pericardial puncture exclusively for biomarker evaluation remains clinically impractical and could be applicable only during open cardiac surgery.

### 8.2. Translational Barriers from Preclinical to Clinical—The Challenge of Adapting Multifactorial Rat Models to Match Human HFpEF Phenotypes

A systematic review conducted by Meijs et al. identified nine distinct HFpEF phenotypes based on their clinically most significant comorbidity, highlighting the potential of using more targeted and personalized therapeutic strategies [95]. Targeted modulation of miRNAs tailored to specific HFpEF subtypes may support the development of such strategies. Multifactorial animal models incorporating aging, hypertension, and metabolic dysfunction replicate HFpEF’s complexity but remain limited in capturing its full clinical heterogeneity [96]. Withaar et al. stated that animal models should replicate key clinical features, including preserved ejection fraction with demonstrable diastolic dysfunction, pulmonary congestion, elevated natriuretic peptides, reduced exercise capacity, and common comorbidities, while also accounting for aging and sex differences, to better reflect the heterogeneity of human HFpEF [97]. However, there is no specific consensus on the specific circulating miRNA panel that should be considered as a definitive biomarker for HFpEF, nor a clear differentiation for their corresponding HFpEF subgroup [98]. Limiting seric miRNA testing to well-defined HFpEF endotypes could enhance novel biomarker discovery by revealing distinct miRNA expression patterns linked to specific pathophysiological mechanisms within each subgroup. Validation analyses in human clinical cohorts should be recommended to substantiate translational relevance and ensure clinical applicability.

### 8.3. Feasibility of Targeted Therapy

Due to their chemical stability in circulation and the sensitivity of current quantification techniques, miRNAs emerge as promising diagnostic biomarkers for assessing inflammatory response severity and as therapeutic targets. Development and application of such approaches should be approached with caution to avoid pathological consequences from excessive modulation, but remain highly promising for diagnosing and treating a wide spectrum of acute and chronic inflammatory diseases [8].

Reversing myocardial fibrosis is an emerging research focus, with yet underexplored pathways—such as intracellular collagen degradation via phagocytosis and collagen cross-linking modulation—offering potential therapeutic pathways [93]. Although some antifibrotic strategies may hold significant clinical potential, their feasibility remains limited [24]. Epigenetic mechanisms, including deoxyribonucleic acid methylation and miRNA regulation, play a critical role in HFpEF development by modulating oxidative stress responses, fibrosis, cardiac hypertrophy, and structural remodeling. However, the precise relationship between these epigenetic changes and distinct HFpEF phenotypes remains incompletely understood [11].

## 9. Conclusions

Substantial diagnostic and therapeutic challenges still exist in HFpEF, with current interventions failing to reverse cardiac remodeling or ameliorate diastolic dysfunction. However, miRNAs emerge as critical regulators within the inflammation–fibrosis cascade, driving HFpEF progression. Specifically, the miRNA network comprising miR-146a, miR-155, miR-223, miR-21, and miR-29 functions as an integrated regulatory system that governs the inflammatory-fibrotic continuum characteristic of HFpEF.

Therapeutic success in HFpEF requires targeting specific molecular pathways, particularly miRNA-mediated circuits, to stop disease progression and restore myocardial homeostasis. This paradigm shift from symptomatic management toward molecular intervention represents the next frontier of HFpEF therapeutics.

## Figures and Tables

**Table 1:** Summary of article selection process for the narrative review.

Step	Description	Number of Articles
Initial search	Articles retrieved from database searches (e.g., PubMed, Scopus, Web of Science) using keywords: “biomarkers”, “HFpEF”, “inflammation”, “fibrosis”, “miRNA”	300
Additional sources	Articles identified through reference lists and relevant review articles	103
Total records identified	Sum of database and manual searches	403
Screening	Titles and abstracts reviewed for relevance to HFpEF and miRNAs	250
Exclusions at Screening	Excluded due to irrelevance, or non-English language	50
Full-text Assessment	Full articles reviewed to confirm inclusion based on relevance, mechanistic detail, and/or miRNA involvement	138
Exclusions after full-text review	Excluded due to insufficient data on HFpEF outcomes or lack of original data	42
Final articles included	Articles included in the narrative review	98

**Table 2:** miRNA expression in HFpEF.

miRNA	Expression Pattern	Main Targets/Pathways	Primary Effects	Role in Inflammation and/or Fibrosis
miR-146a	Upregulated	NF-κB pathway components via IRAK1, TRAF6	Reduces/enhances NF-κB activation	Pro- and anti-inflammatory; antifibrotic
miR-155	Upregulated	M1 macrophage polarization, ROS synthesis	Promotes pro-inflammatory gene expression	Pro-inflammatory
miR-223	Downregulated	NF-κB activation	Suppresses neutrophil activation and chemotaxis	Pro-inflammatory
miR-21	Upregulated	TGF-β/Smad, SPRY1/ERK-MAP pathways	Promotes fibroblast survival, ECM production	Pro- and anti-inflammatory; profibrotic
miR-29	Downregulated	ECM proteins (collagen I/III, fibrillin, elastin), TGFβ2, MMP2	Limits ECM production	Anti-inflammatory; antifibrotic
miR-208a/b	Upregulated	Endoglin, collagen I	Promotes myofibroblast differentiation, hypertrophy	Profibrotic
miR-133a	Downregulated	Collagen1A1	Regulates sarcomere formation, cardiomyocyte structure	Antifibrotic

## Data Availability

No new data were created or analyzed in this study.

## Abbreviations

HF—heart failure; HFpEF—heart failure with preserved ejection fraction; HFrEF—heart failure with reduced ejection fraction; miRNAs—micro ribonucleic acids; IL-1β—interleukin 1β; TNF-α—tumor necrosis factor-α, and IL-6—interleukin 6; MAPKs—mitogen-activated protein kinases; NF-κB—nuclear factor kappa-light-chain-enhancer of activated B cells; ECM—extracellular matrix; TNFR- tumor necrosis factor receptors; VCAM—vascular cell adhesion molecules; TGF-β—transforming growth factor-β; ROS—reactive oxygen species; UPR—unfolded protein response; DAMP—damage-associated molecular patterns; TLR—toll-like receptors; RAAS—renin–angiotensin–aldosterone system; AT1R—angiotensin II type 1 receptors; IRAK1—interleukin-1-associated-kinase-1; Myd88—primary response protein 88; ERK—extracellular signal-regulated kinase; MMP2—matrix metalloproteinase 2; α-MHC—α-isoform of myosin heavy chain; PI3K/Akt—phosphoinositide 3-kinase/protein kinase B; ASO—antisense oligonucleotides; siRNA—small interfering RNAs; SGLT2i sodium-glucose cotransporter 2 inhibitors.

## References

[1] Daubert C. (2024). Heart failure: A major public health problem. La Presse Medicale.

[2] Dunlay S.M., Roger V.L., Redfield M.M. (2017). Epidemiology of heart failure with preserved ejection fraction. Nat. Rev. Cardiol..

[3] Fletcher R.A., Rockenschaub P., Neuen B.L., Walter I.J., Conrad N., Mizani M.A., Bolton T., Lawson C.A., Tomlinson C., Logothetis S.B. (2024). Contemporary epidemiology of hospitalised heart failure with reduced versus preserved ejection fraction in England: A retrospective, cohort study of whole-population electronic health records. Lancet Public Health.

[4] Teramoto K., Teng T.H.K., Chandramouli C., Tromp J., Sakata Y., Lam C.S.P. (2022). Epidemiology and Clinical Features of Heart Failure with Preserved Ejection Fraction. Card. Fail. Rev..

[5] Omote K., Verbrugge F.H., Borlaug B.A. (2022). Heart Failure with Preserved Ejection Fraction: Mechanisms and Treatment Strategies. Annu. Rev. Med..

[6] Paraskevaidis I., Farmakis D., Papingiotis G., Tsougos E. (2023). Inflammation and Heart Failure: Searching for the Enemy—Reaching the Entelechy. J. Cardiovasc. Dev. Dis..

[7] Orang A.V., Safaralizadeh R., Kazemzadeh-Bavili M. (2014). Mechanisms of miRNA-mediated gene regulation from common downregulation to mRNA-specific upregulation. Int. J. Genom..

[8] Das K., Rao L.V.M. (2022). The Role of microRNAs in Inflammation. Int. J. Mol. Sci..

[9] Cai Y., Yu X., Hu S., Yu J. (2009). A Brief Review on the Mechanisms of miRNA Regulation. Genom. Proteom. Bioinform..

[10] Oliveira-Carvalho V., Carvalho V.O., Silva M.M., Guimarães G.V., Bocchi E.A. (2012). MicroRNAs: A new paradigm in the treatment and diagnosis of heart failure?. Arq. Bras. De Cardiol..

[11] Berezin A.E. (2016). Epigenetic Modifications the Development of Different Heart Failure Phenotypes. J. Data Min. Genom. Proteom..

[12] Tschöpe C., Van Linthout S. (2014). New insights in (inter) cellular mechanisms by heart failure with preserved ejection fraction. Curr. Heart Fail. Rep..

[13] Van Linthout S., Tschöpe C. (2017). Inflammation—Cause or Consequence of Heart Failure or Both?. Curr. Heart Fail. Rep..

[14] Prabhu S.D., Frangogiannis N.G. (2016). The biological basis for cardiac repair after myocardial infarction. Circ. Res..

[15] Frati G., Schirone L., Chimenti I., Yee D., Biondi-Zoccai G., Volpe M., Sciarretta S. (2017). An overview of the inflammatory signaling mechanisms in the myocardium underlying the development of diabetic cardiomyopathy. Cardiovasc. Res..

[16] Bartekova M., Radosinska J., Jelemensky M., Dhalla N.S. (2018). Role of cytokines and inflammation in heart function during health and disease. Heart Fail. Rev..

[17] Rose N.R. (2011). Critical cytokine pathways to cardiac inflammation. J. Interferon Cytokine Res..

[18] Mihara M., Hashizume M., Yoshida H., Suzuki M., Shiina M. (2012). IL-6/IL-6 receptor system and its role in physiological and pathological conditions. Clin. Sci..

[19] Sanders-Van Wijk S., Tromp J., Beussink-Nelson L., Hage C., Svedlund S., Saraste A., Swat S.A., Sanchez C., Njoroge J., Tan R.-S. (2020). Proteomic Evaluation of the Comorbidity-Inflammation Paradigm in Heart Failure With Preserved Ejection Fraction Results From the PROMIS-HFpEF Study. Circulation.

[20] Hage C., Michaëlsson E., Linde C., Donal E., Daubert J.C., Gan L.M., Lund L.H. (2017). Inflammatory Biomarkers Predict Heart Failure Severity and Prognosis in Patients with Heart Failure with Preserved Ejection Fraction: A Holistic Proteomic Approach. Circ. Cardiovasc. Genet..

[21] Putko B.N., Wang Z., Lo J., Anderson T., Becher H., Dyck J.R.B., Kassiri Z., Oudit G.Y., Calvert J., Alberta HEART Investigators (2014). Circulating levels of tumor necrosis factor-alpha receptor 2 are increased in heart failure with preserved ejection fraction relative to heart failure with reduced ejection fraction: Evidence for a divergence in pathophysiology. PLoS ONE.

[22] Carris N.W., Mhaskar R., Coughlin E., Bracey E., Tipparaju S.M., Halade G.V. (2022). Novel biomarkers of inflammation in heart failure with preserved ejection fraction: Analysis from a large prospective cohort study. BMC Cardiovasc. Disord..

[23] Souders C.A., Bowers S.L.K., Baudino T.A. (2009). Cardiac fibroblast: The renaissance cell. Circ. Res..

[24] Frangogiannis N.G. (2021). Cardiac fibrosis. Cardiovasc. Res..

[25] Sweeney M., Corden B., Cook S.A. (2020). Targeting cardiac fibrosis in heart failure with preserved ejection fraction: Mirage or miracle?. EMBO Mol. Med..

[26] Gullestad L., Ueland T., Vinge L.E., Finsen A., Yndestad A., Aukrust P. (2012). Inflammatory cytokines in heart failure: Mediators and markers. Cardiology.

[27] Paulus W.J., Zile M.R. (2021). From Systemic Inflammation to Myocardial Fibrosis: The Heart Failure with Preserved Ejection Fraction Paradigm Revisited. Circ. Res..

[28] Cazorla O., Freiburg A., Helmes M., Centner T., McNabb M., Wu Y., Trombitas K., Labeit S., Granzier H. (2000). Differential expression of cardiac titin isoforms and modulation of cellular stiffness. Circ. Res..

[29] Dominic K.L., Schmidt A.V., Granzier H., Campbell K.S., Stelzer J.E. (2024). Mechanism-based myofilament manipulation to treat diastolic dysfunction in HFpEF. Front. Physiol..

[30] Valée A., Lecarpentier Y. (2019). TGF-β in fibrosis by acting as a conductor for contractile properties of myofibroblasts. Cell Biosci..

[31] Khalaji A., Mehrtabar S., Jabraeilipour A., Doustar N., Rahmani Youshanlouei H., Tahavvori A., Fattahi P., Alavi S.M., Taha S.R., Fazlollahpour-Naghibi A. (2024). Inhibitory effect of microRNA-21 on pathways and mechanisms involved in cardiac fibrosis development. Ther. Adv. Cardiovasc. Dis..

[32] Doğan A.Ş., Pala M., Görücü Yilmaz Ş., Beceren A., Karabulut A., Polat Y., Elçioğlu H.K. (2025). The Effects of MicroRNAs on Cardiomyopathy in a Rat Model of Streptozotocin-induced Diabetes Mellitus. Bezmialem Sci..

[33] Bonanni A., Vinci R., d’Aiello A., Grimaldi M.C., Di Sario M., Tarquini D., Proto L., Severino A., Pedicino D., Liuzzo G. (2023). Targeting collagen pathways as an HFpEF therapeutic strategy. J. Clin. Med..

[34] Kabłak-Ziembicka A., Badacz R., Okarski M., Wawak M., Przewłocki T., Podolec J. (2023). Cardiac microRNAs: Diagnostic and therapeutic potential. Arch. Med. Sci..

[35] Olivieri F., Prattichizzo F., Giuliani A., Matacchione G., Rippo M.R., Sabbatinelli J., Bonafè M. (2021). miR-21 and miR-146a: The microRNAs of inflammaging and age-related diseases. Ageing Res. Rev..

[36] Feng B., Chen S., Gordon A.D., Chakrabarti S. (2017). miR-146a mediates inflammatory changes and fibrosis in the heart in diabetes. J. Mol. Cell. Cardiol..

[37] Gholaminejad A., Zare N., Dana N., Shafie D., Mani A., Javanmard S.H. (2021). A meta-analysis of microRNA expression profiling studies in heart failure. Heart Fail. Rev..

[38] Shimada B.K., Yang Y., Zhu J., Wang S., Suen A., Kronstadt S.M., Jeyaram A., Jay S.M., Zou L., Chao W. (2020). Extracellular miR-146a-5p Induces Cardiac Innate Immune Response and Cardiomyocyte Dysfunction. Immunohorizons.

[39] Dueck A., Eichner A., Sixt M., Meister G. (2014). A miR-155-dependent microRNA hierarchy in dendritic cell maturation and macrophage activation. FEBS Lett..

[40] Hu J., Huang S., Liu X., Zhang Y., Wei S., Hu X. (2022). miR-155: An important role in inflammation response. J. Immunol. Res..

[41] Pasca S., Jurj A., Petrushev B., Tomuleasa C., Matei D. (2020). MicroRNA-155 Implication in M1 Polarization and the Impact in Inflammatory Diseases. Front. Immunol..

[42] Jablonski K.A., Gaudet A.D., Amici S.A., Popovich P.G., Guerau-de-Arellano M. (2016). Control of the inflammatory macrophage transcriptional signature by miR-155. PLoS ONE.

[43] Yuan X., Berg N., Lee J.W., Le T.T., Neudecker V., Jing N., Eltzschig H. (2018). MicroRNA miR-223 as regulator of innate immunity. J. Leukoc. Biol..

[44] Johnnidis J.B., Harris M.H., Wheeler R.T., Stehling-Sun S., Lam M.H., Kirak O., Brummelkamp T.R., Fleming M.D., Camargo F.D. (2008). Regulation of progenitor cell proliferation and granulocyte function by microRNA-223. Nature.

[45] Taïbi F., Metzinger-Le Meuth V., Massy Z.A., Metzinger L. (2014). miR-223: An inflammatory oncomiR enters the cardiovascular field. Biochim. Biophys. Acta—Mol. Basis Dis..

[46] Haneklaus M., Gerlic M., O’Neill L.A.J., Masters S.L. (2013). miR-223: Infection, inflammation and cancer. J. Intern. Med..

[47] Liu X., Xu Y., Deng Y., Li H. (2018). MicroRNA-223 Regulates Cardiac Fibrosis after Myocardial Infarction by Targeting RASA1. Cell. Physiol. Biochem..

[48] Yuan J., Chen H., Ge D., Xu Y., Xu H., Yang Y., Gu M., Zhou Y., Zhu J., Ge T. (2017). miR-21 Promotes Cardiac Fibrosis after Myocardial Infarction Via Targeting Smad7. Cell. Physiol. Biochem..

[49] Surina Fontanella R.A., Scisciola L., Marfella R., Paolisso G., Barbieri M. (2021). miR-21 in human cardiomyopathies. Front. Cardiovasc. Med..

[50] Cardin S., Guasch E., Luo X., Naud P., Le Quang K., Shi Y., Tardif J.-C., Comtois P., Nattel S. (2012). Role for MicroRNA-21 in atrial profibrillatory fibrotic remodeling associated with experimental postinfarction heart failure. Circ. Arrhythm. Electrophysiol..

[51] Ben-Nun D., Buja L.M., Fuentes F. (2020). Prevention of heart failure with preserved ejection fraction (HFpEF): Reexamining microRNA-21 inhibition in the era of oligonucleotide-based therapeutics. Cardiovasc. Pathol..

[52] Dong D.L., Yang B.F. (2011). Role of microRNAs in cardiac hypertrophy, myocardial fibrosis and heart failure. Acta Pharm. Sin. B.

[53] Vegter E.L., Van Der Meer P., De Windt L.J., Pinto Y.M., Voors A.A. (2016). MicroRNAs in heart failure: From biomarker to target for therapy. Eur. J. Heart Fail..

[54] Shen N.N., Wang J.L., Fu Y.P. (2022). The microRNA expression profiling in heart failure: A systematic review and meta-analysis. Front. Cardiovasc. Med..

[55] Chang W.T., Shih J.Y., Lin Y.W., Huang T.L., Chen Z.C., Chen C.L., Chu J.S., Liu P.Y. (2022). miR-21 upregulation exacerbates pressure overload-induced cardiac hypertrophy in aged hearts. Aging.

[56] Kura B., Kalocayova B., Devaux Y., Bartekova M. (2020). Potential clinical implications of miR-1 and miR-21 in heart disease and cardioprotection. Int. J. Mol. Sci..

[57] Shi P., Zhao X.D., Shi K.H., Ding X.S., Tao H. (2021). miR-21–3p triggers cardiac fibroblasts pyroptosis in diabetic cardiac fibrosis via inhibiting androgen receptor. Exp. Cell Res..

[58] Dalgaard L.T., Sørensen A.E., Hardikar A.A., Joglekar M.V. (2022). The microRNA-29 family: Role in metabolism and metabolic disease. American journal of physiology. Cell Physiol..

[59] Van Rooij E., Sutherland L.B., Thatcher J.E., DiMaio J.M., Naseem R.H., Marshall W.S., Hill J.A., Olson E.N. (2008). Dysregulation of microRNAs after myocardial infarction reveals a role of miR-29 in cardiac fibrosis. Proc. Natl. Acad. Sci. USA.

[60] Li C., Wang N., Rao P., Wang L., Lu D., Sun L. (2021). Role of the microRNA-29 family in myocardial fibrosis. J. Physiol. Biochem..

[61] Zhang X., McLendon J.M., Peck B.D., Chen B., Song L.S., Boudreau R.L. (2023). Modulation of miR-29 influences myocardial compliance likely through coordinated regulation of calcium handling and extracellular matrix. Mol. Ther. Nucleic Acids.

[62] Zhou H., Tang W., Yang J., Peng J., Guo J., Fan C. (2021). MicroRNA-related strategies to improve cardiac function in heart failure. Front. Cardiovasc. Med..

[63] Liu M.N., Luo G., Gao W.J., Yang S.J., Zhou H. (2021). miR-29 family: A potential therapeutic target for cardiovascular disease. Pharmacol. Res..

[64] Huang X.H., Li J.L., Li X.Y., Wang S.X., Jiao Z.H., Li S.Q., Liu J., Ding J. (2021). miR-208a in cardiac hypertrophy and remodeling. Front. Cardiovasc. Med..

[65] Dickinson B.A., Semus H.M., Montgomery R.L., Stack C., Latimer P.A., Lewton S.M., Lynch J.M., Hullinger T.G., Seto A.G., van Rooij E. (2013). Plasma microRNAs serve as biomarkers of therapeutic efficacy and disease progression in hypertension-induced heart failure. Eur. J. Heart Fail..

[66] Zhang X.T., Xu M.G. (2021). Potential link between microRNA-208 and cardiovascular diseases. J. Xiangya Med..

[67] Castoldi G., di Gioia C.R.T., Bombardi C., Catalucci D., Corradi B., Gualazzi M.G., Leopizzi M., Mancini M., Zerbini G., Condorelli G. (2012). miR-133a regulates collagen 1A1: Potential role of miR-133a in myocardial fibrosis in angiotensin II-dependent hypertension. J. Cell. Physiol..

[68] Kruk L., Braun A., Cosset E., Gudermann T., Mammadova-Bach E. (2023). Galectin functions in cancer-associated inflammation and thrombosis. Front. Cardiovasc. Med..

[69] Wang J., Han B. (2020). Dysregulated CD4+ T cells and microRNAs in myocarditis. Front. Immunol..

[70] Sygitowicz G., Maciejak-Jastrzębska A., Sitkiewicz D. (2021). The diagnostic and therapeutic potential of galectin-3 in cardiovascular diseases. Biomolecules.

[71] Gareev I., Beylerli O., Sufianov A., Gulieva L., Pavlov V., Shi H. (2025). MicroRNAs in the Regulation of Immune Response in Cardiovascular Diseases: New Diagnostic and Therapeutic Tools. Gene Expr..

[72] Chen Y., Ye X., Escames G., Lei W., Zhang X., Li M., Jing T., Yao Y., Qiu Z., Wang Z. (2023). The NLRP3 inflammasome: Contributions to inflammation-related diseases. Cell. Mol. Biol. Lett..

[73] Chen T., Li Z., Tu J., Zhu W., Ge J., Zheng X., Yang L., Pan X., Yan H., Zhu J. (2011). MicroRNA-29a regulates pro-inflammatory cytokine secretion and scavenger receptor expression by targeting LPL in oxLDL-stimulated dendritic cells. FEBS Lett..

[74] Sansonetti M., De Windt L.J. (2022). Non-coding RNAs in cardiac inflammation: Key drivers in the pathophysiology of heart failure. Cardiovasc. Res..

[75] Hassanabad A.F., Zarzycki A.N., Patel V.B., Fedak P.W. (2024). Current concepts in the epigenetic regulation of cardiac fibrosis. Cardiovasc. Pathol..

[76] Zhang J., Xing Q., Zhou X., Li J., Li Y., Zhang L., Zhou Q., Tang B. (2017). Circulating miRNA-21 is a promising biomarker for heart failure. Mol. Med. Rep..

[77] Wang L., Guo A., Liang S., Yu L., Shen B., Huang Z. (2025). The association of serum hsa-miR-21-5p expression with the severity and prognosis of heart failure with reduced ejection fraction. BMC Cardiovasc. Disord..

[78] Yamada H., Suzuki K., Fujii R., Kawado M., Hashimoto S., Watanabe Y., Iso H., Fujino Y., Wakai K., Tamakoshi A. (2021). Circulating miR-21, miR-29a, and miR-126 are associated with premature death risk due to cancer and cardiovascular disease: The JACC Study. Sci. Rep..

[79] Veitch S., Njock M.S., Chandy M., Siraj M.A., Chi L., Mak H., Yu K., Rathnakumar K., Perez Romero C.A., Chen Z. (2022). miR-30 promotes fatty acid beta-oxidation and endothelial cell dysfunction and is a circulating biomarker of coronary microvascular dysfunction in pre-clinical models of diabetes. Cardiovasc. Diabetol..

[80] Kattih B., Fischer A., Muhly-Reinholz M., Tombor L., Nicin L., Cremer S., Zeiher A.M., John D., Abplanalp W.T., Dimmeler S. (2025). Inhibition of miR-92a normalizes vascular gene expression and prevents diastolic dysfunction in heart failure with preserved ejection fraction. J. Mol. Cell. Cardiol..

[81] Couch L.S., Fiedler J., Chick G., Clayton R., Dries E., Wienecke L.M., Fu L., Fourre J., Pandey P., Derda A.A. (2022). Circulating microRNAs predispose to takotsubo syndrome following high-dose adrenaline exposure. Cardiovasc. Res..

[82] Wong L.L., Zou R., Zhou L., Lim J.Y., Phua D.C., Liu C., Chong J.P., Ng J.Y., Liew O.W., Chan S.P. (2019). Combining Circulating MicroRNA and NT-proBNP to Detect and Categorize Heart Failure Subtypes. J. Am. Coll. Cardiol..

[83] Arul J.C., Raja Beem S.S., Parthasarathy M., Kuppusamy M.K., Rajamani K., Silambanan S. (2025). Association of microRNA-210-3p with NT-proBNP, sST2, and Galectin-3 in heart failure patients with preserved and reduced ejection fraction: A cross-sectional study. PLoS ONE.

[84] Parvan R., Hosseinpour M., Moradi Y., Devaux Y., Cataliotti A., da Silva G.J.J. (2022). Diagnostic performance of microRNAs in the detection of heart failure with reduced or preserved ejection fraction: A systematic review and meta-analysis. Eur. J. Heart Fail..

[85] Zhang M.W., Shen Y.J., Shi J., Yu J.G. (2021). miR-223-3p in cardiovascular diseases: A biomarker and potential therapeutic target. Front. Cardiovasc. Med..

[86] D’Amato A., Prosperi S., Severino P., Myftari V., Correale M., Perrone Filardi P., Badagliacca R., Fedele F., Vizza C.D., Palazzuoli A. (2024). MicroRNA and Heart Failure: A Novel Promising Diagnostic and Therapeutic Tool. J. Clin. Med..

[87] Traber G.M., Yu A.-M. (2023). RNAi-based therapeutics and novel RNA bioengineering technologies. J. Pharmacol. Exp. Ther..

[88] Montgomery R.L., Hullinger T.G., Semus H.M., Dickinson B.A., Seto A.G., Lynch J.M., Stack C., Latimer P.A., Olson E.N., Van Rooij E. (2011). Therapeutic inhibition of miR-208a improves cardiac function and survival during heart failure. Circulation.

[89] Bertaud A., Joshkon A., Heim X., Bachelier R., Bardin N., Leroyer A.S., Blot-Chabaud M. (2023). Signaling pathways and potential therapeutic strategies in cardiac fibrosis. Int. J. Mol. Sci..

[90] McDonagh T.A., Metra M., Adamo M., Gardner R.S., Baumbach A., Böhm M., Burri H., Butler J., Čelutkienė J., Chioncel O. (2023). 2023 focused update of the 2021 ESC guidelines for the diagnosis and treatment of acute and chronic heart failure: Developed by the task force for the diagnosis and treatment of acute and chronic heart failure of the European Society of Cardiology (ESC) with the special contribution of the Heart Failure Association (HFA) of the ESC. Eur. Heart J..

[91] Chen Y., Peng D. (2023). New insights into the molecular mechanisms of SGLT2 inhibitors on ventricular remodeling. Int. Immunopharmacol..

[92] Zhang Y., Lin X., Chu Y., Chen X., Du H., Zhang H., Xu C., Xie H., Ruan Q., Lin J. (2021). Dapagliflozin: A sodium–glucose cotransporter 2 inhibitor, attenuates angiotensin II-induced cardiac fibrotic remodeling by regulating TGFβ1/Smad signaling. Cardiovasc. Diabetol..

[93] Schimmel K., Ichimura K., Reddy S., Haddad F., Spiekerkoetter E. (2022). Cardiac fibrosis in the pressure overloaded left and right ventricle as a therapeutic target. Front. Cardiovasc. Med..

[94] Schoettler F.I., Hassanabad A.F., Jadli A.S., Patel V.B., Fedak P.W. (2024). Exploring the role of pericardial miRNAs and exosomes in modulating cardiac fibrosis. Cardiovasc. Pathol..

[95] Meijs C., Handoko M.L., Savarese G., Vernooij R.W.M., Vaartjes I., Banerjee A., Koudstaal S., Brugts J.J., Asselbergs F.W., Uijl A. (2023). Discovering Distinct Phenotypical Clusters in Heart Failure Across the Ejection Fraction Spectrum: A Systematic Review. Curr. Heart Fail. Rep..

[96] Gao S., Liu X.P., Li T.T., Chen L., Feng Y.P., Wang Y.K., Yin Y.J., Little P.J., Wu X.Q., Xu S.W. (2024). Animal models of heart failure with preserved ejection fraction (HFpEF): From metabolic pathobiology to drug discovery. Acta Pharmacol. Sin..

[97] Withaar C., Lam C.S.P., Schiattarella G.G., De Boer R.A., Meems L.M.G. (2021). Heart failure with preserved ejection fraction in humans and mice: Embracing clinical complexity in mouse models. Eur. Heart J..

[98] Bayes-Genis A., Cediel G., Domingo M., Codina P., Santiago E., Lupón J. (2022). Biomarkers in heart failure with preserved ejection fraction. Card. Fail. Rev..

